# Referral to cancer rehabilitation in the cancer care continuum: barriers, inequities, and opportunities for integration—a scoping review

**DOI:** 10.3389/fpsyg.2026.1918643

**Published:** 2026-09-24

**Authors:** Khawla Loubani, Katie M. Polo, Taylor Brown, Korrin Schalhamer, Alexandria Gilley, Morgan Jones, Cara Murphy, Olivia Witteborg, Rachel Graves

**Affiliations:** 1 Department of Occupational Therapy, Faculty of Health Sciences, Ben-Gurion University of the Negev, Beer-Sheva, Israel; 2 School of Occupational Therapy, College of Health Sciences, University of Indianapolis, Indianapolis, IN, United States

**Keywords:** cancer rehabilitation, rehabilitation referral, healthcare professionals, clinician perceptions, rehabilitation knowledge, interdisciplinary coordination, functional needs, health inequities

## Abstract

**Background:**

Functional impairments among people living with and beyond cancer can affect treatment tolerance, daily functioning, participation, and quality of life. Despite the potential benefits of rehabilitation, referral remains inconsistent. Referral barriers may also arise within related supportive-care pathways, particularly palliative care, although the purposes and indications of these services differ.

**Objective:**

This scoping review aimed to synthesize evidence on facilitators and barriers affecting referral across two related but conceptually distinct service contexts: palliative care and cancer rehabilitation or allied health services.

**Methods:**

A comprehensive search of major electronic databases and gray literature sources was conducted for studies published in English between January 2011 and January 2025. Eligible studies examined factors affecting referral to cancer rehabilitation or allied health services or addressed palliative care referral when rehabilitation-relevant factors were reported. Two authors independently screened the studies and appraised their evidence level and methodological quality using the Johns Hopkins Evidence-Based Practice Research Evidence Appraisal Tool. Findings were synthesized thematically and stratified by service context.

**Results:**

Eighteen studies were included: eight examined referral to palliative care and ten examined cancer rehabilitation, allied health services, or rehabilitation screening and triage pathways. The evidence consisted of 12 quantitative, three qualitative, and three mixed-methods studies and was predominantly Level III. Four themes were identified: clinicians' perceptions of referral and services; the influence of social determinants of health; individual cancer characteristics and demographics; and interdisciplinary coordination, communication, and education. In palliative care, referral was commonly associated with advanced disease, symptom burden, uncertainty regarding referral timing, and misconceptions about the purpose of palliative care. In rehabilitation and allied health services, referral was more dependent on recognition of functional needs, clinicians' knowledge of rehabilitation roles and available services, and the presence of clear referral pathways. Limited interdisciplinary coordination, clinician-dependent decision-making, and inequities in access were evident across both contexts, although their manifestations differed.

**Conclusions:**

Referral barriers should be interpreted within the distinct clinical purposes and pathways of palliative care and cancer rehabilitation. Structured, context-specific referral pathways may support more consistent access. Within cancer rehabilitation, routine functional screening, explicit referral criteria, and stronger integration of rehabilitation professionals into oncology teams warrant further implementation and evaluation.

## Introduction

Progress in cancer treatment has resulted in a rapidly growing population of people living with and beyond cancer (PLWBC), many of whom experience persistent functional and cognitive impairments. Functional impairment is a prevalent and often underrecognized consequence of cancer and its treatment (Neo et al., 2017; Siegel et al., 2020). Across the disease trajectory, PLWBC may experience a range of cancer- and treatment-related impairments, including fatigue, neuropathy, cognitive dysfunction, deconditioning, and psychosocial distress, which may compromise independence and participation (Loh and Jonsson, 2016). These impairments extend beyond quality-of-life concerns; they influence treatment tolerance, healthcare utilization, and long-term survivorship outcomes (Dal Maso et al., 2022; Emery et al., 2022). As global cancer survivorship increases, preserving functional capacity has become central to high-quality care, including referral to cancer rehabilitation (Egger-Heidrich et al., 2025).

However, despite increasing evidence supporting rehabilitation, referral to cancer rehabilitation services remains inconsistent across cancer care pathways. Referral practices may also vary across healthcare systems because of differences in service organization, funding and reimbursement, availability of rehabilitation professionals, and the presence or absence of standardized referral pathways. These differences should be considered when comparing referral patterns across countries (Loubani et al., 2024, 2026). Various factors can affect referrals for PLWBC, including organizational procedures, interdisciplinary communication, coordination among healthcare professionals, and understanding the roles of rehabilitation professionals (Loubani et al., 2024; Mackenzie, 2021; Olsson Möller et al., 2020).

Limited access to needed allied health rehabilitation services may contribute to reduced functional recovery, lower quality of life, and decreased treatment tolerance. Existing studies indicate underutilization of specific cancer rehabilitation services; however, the reported estimates were derived from different populations, denominators, healthcare systems, service models, and definitions of referral or utilization. Pergolotti et al. (2014) found that only 32% of older cancer survivors who were identified as needing OT received the service within the first 2 years of their cancer diagnosis. This finding concerned utilization of an individual rehabilitation discipline rather than access to a comprehensive multidisciplinary cancer rehabilitation program. In a cross-sectional survey of 57 physical therapists in Saudi Arabia, Abdelbasset et al. (2023) found that only 13 (22.8%) respondents reported receiving referrals of patients with breast cancer from clinicians or oncologists to the PT departments in which they worked. This estimate represented therapists' reports of referral practices and not a population-based referral rate among patients with breast cancer. Similarly, Polo et al. (2022) found that, among participants with various cancer diagnoses who reported moderate-to-severe functional impairments, only 29% had ever been referred to needed OT services. These studies indicate gaps in access to individual rehabilitation services, which are increasingly recognized as a core component of comprehensive cancer survivorship care (Braveman and Robin, 2020; Manne and Nekhlyudov, 2024). However, their estimates should not be interpreted as directly comparable measures of access to comprehensive cancer rehabilitation.

Cancer rehabilitation is a multidisciplinary person-centered process aimed at maintaining, restoring or optimizing physical, cognitive, psychological functioning, participation and quality of life affected by cancer and its treatments. It is relevant across all phases of the cancer continuum and may be delivered through comprehensive multidisciplinary programs or through referral to individual rehabilitation disciplines (Loubani et al., 2022; Pergolotti et al., 2019). Comprehensive cancer rehabilitation may involve rehabilitation medicine, occupational therapy (OT), physical therapy (PT), speech-language therapy, exercise-based rehabilitation, psycho-oncology, nutritional counseling, and social and vocational rehabilitation. In this review, referrals to comprehensive multidisciplinary programs and referrals to individual rehabilitation services were considered related but distinct service models.

Within this continuum-based framework, rehabilitation may also be provided throughout palliative care, including within general and palliative care services. Palliative care is not limited to hospice or end-of-life care; it may be provided alongside active cancer treatment and aims to minimize treatment- and disease-related symptoms while maximizing comfort and quality of life (Braveman and Robin, 2020). These aims overlap with those of palliative rehabilitation, which seeks to maintain function, participation, independence, and quality of life despite progressive disease. Accordingly, this review considered palliative rehabilitation an integral component of the cancer rehabilitation continuum. Therefore this scoping review aimed to map and synthesize available evidence on multilevel facilitators and barriers affecting referral to allied health rehabilitation services across the cancer care continuum, including palliative care. The findings may inform future clinical and policy efforts to improve multidisciplinary cancer care coordination. The review questions were:

(1) What evidence is available regarding referral to cancer rehabilitation services and related palliative care pathways across the cancer continuum?
(2) What factors (facilitators/barriers) influence these referrals, and how do the reported factors vary across rehabilitation and palliative care service models?

## Material and methods

### Data extraction and synthesis

This study conforms to all Preferred Reporting Items for Systematic Reviews and Meta-Analyses Extension for Scoping Reviews (PRISMA-ScR) (Tricco et al., 2018) guidelines and reports the required information accordingly. The PRISMA flowchart (Figure 1) outlines the selection process.

**Figure 1:**
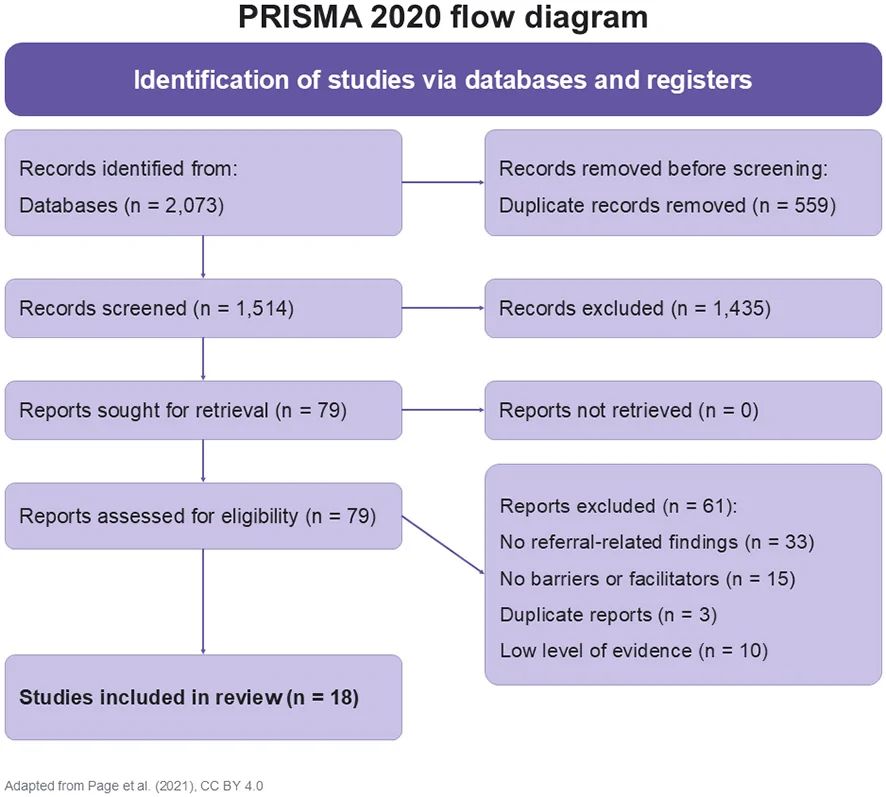
PRISMA 2020 flow diagram of the study-selection process. Adapted from Page et al. (2021) under the Creative Commons Attribution 4.0 International License (CC BY 4.0). Source: https://www.prisma-statement.org/prisma-2020-flow-diagram.

We conducted a scoping review because this methodology is an ideal tool for assessing the scope and volume of the literature on a given topic, providing a clear overview of its focus. This approach helps identify available evidence types, clarify key concepts, and analyze knowledge gaps (Munn et al., 2018). We followed the methodology described by Arksey and O'Malley (2005) and searched for articles published between January 2011 and January 2025. The review process involved identifying the population, intervention, comparison, outcome, and time question; searching for relevant studies (including gray literature); selecting and reviewing the identified studies; charting data from the studies (e.g., author, publication year, country, intervention type, study population, aims, methods, outcomes, and results); and analyzing, summarizing, and reporting the findings (Arksey and O'Malley, 2005; Cacchione, 2016). Our review focused on issues related to cancer rehabilitation referrals and the factors influencing them. Because the literature has not always clearly distinguished palliative rehabilitation from broader palliative care referral, particularly in studies of referral pathways for people with advanced cancer, studies examining palliative care referral were also included when they addressed rehabilitation-relevant factors such as symptom burden, functional status, interdisciplinary coordination, service access, or maintenance of quality of life. However, these studies were identified as palliative care studies, and their findings were distinguished from evidence concerning referral to individual allied health services or comprehensive multidisciplinary rehabilitation programs.

The review was reported in accordance with the PRISMA-ScR reporting guideline (Tricco et al., 2018). Ethical approval was not required.

### Eligibility criteria

Studies were eligible if they: (a) involved PLWBC and/or healthcare professionals; (b) were published in English between 2011 and 2025; (c) explicitly addressed facilitators and/or barriers to cancer rehabilitation referral or to palliative care referral when rehabilitation-relevant factors were examined; and (d) were classified as Levels I to III according to the Johns Hopkins Evidence-Based Practice Model (Crawford, 2009). This prespecified restriction was applied to prioritize empirical evidence derived from experimental, quasi-experimental, nonexperimental, qualitative, and mixed-methods research. The exclusion criteria were articles not written in English or published before 2011.

We conducted a gray literature search using the PROSPERO and Trip databases. In collaboration with an expert in scoping reviews, who approved the *a priori* list of search terms and databases, we conducted a faceted search across the main indexed databases for rehabilitation, including PubMed, CINAHL, Academic Search Complete, MEDLINE, and PsycINFO. Further, a manual search complemented the original Medline searches. Using the controlled vocabulary lists of the identified databases, each term was searched for the primary term “referral” and the subterms “cancer,” “rehabilitation,” and “medical professionals” (OT, PT, speech-language therapy, nursing, social work, physician, and oncologist). Filters, such as “date range,” “language,” and “subject heading,” were used to refine the search. We extracted data to compare and analyze the outcomes, identifying and managing duplicate articles using Zotero (Corporation for Digital Scholarship, Falls Church, VA, United States). We then transferred the articles into the Rayyan software and sorted them according to exclusion and inclusion criteria (Ouzzani et al., 2016). Two independent authors reviewed all citations and abstracts. After unblinding, they compared the included studies and discussed differences. In the event of disagreement, a third author was consulted to reach a final decision. At least two authors conducted a full-text review of the accepted citations (*n* = 79). Two authors then independently appraised the evidence level and methodological quality of each included study using the Johns Hopkins Evidence-Based Practice Research Evidence Appraisal Tool. Following the independent appraisals, the authors met to discuss any discrepancies and reached consensus on the final evidence levels and quality grades. Evidence level and methodological quality were recorded as distinct dimensions. Quantitative and mixed-methods studies were assigned a quality grade of A, B, or C, whereas qualitative studies were classified as A/B (high/good quality) or C (low quality). This appraisal was used to characterize the methodological quality of the included evidence. After individually synthesizing each article's findings, we followed Arksey and O'Malley (2005) framework to examine overall findings across articles and to establish dominant themes as the key research and influential factors in referral. During this process, we conducted a preliminary thematic analysis and discussed the findings until we reached consensus on the final themes.

## Results

Our database search of articles published from January 2011 to January 2025 yielded 2,073 articles, of which 559 were duplicates. After screening duplicates and relevance and applying the eligibility criteria, 18 studies were included in the final review. Articles were stratified into two categories: (1) palliative care and (2) allied health rehabilitation services. Eight studies examined referral to palliative care, whereas 10 examined allied health rehabilitation services (cancer rehabilitation, individual allied health services, or rehabilitation screening and triage pathways).

Table 1 summarizes the characteristics of the 18 included studies and identifies the referral or service context of each study. The studies used qualitative (*n* = 3), quantitative (*n* = 12), and mixed-methods (*n* = 3) designs, with sample sizes ranging from 28 to 204,175 participants. Eight studies were conducted in the United States, two in Denmark, two in Canada, and one each in Australia, Brazil, Germany, Israel, Uganda, and the United Kingdom. Most studies were classified as Level III evidence.

**Table 1:** Characteristics of the included studies, stratified by service context.

References	Service context	Study methodology	Population, sample size, and setting	Main objective	Main findings relevant to referral	Conclusion and implications	Country	Evidence level/quality grade^a^
Feld et al. (2019)	Palliative care	Quantitative cross-sectional survey	Medical oncologists treating advanced metastatic non-small-cell lung cancer (*n* = 279 invited; *n* = 93 responded) and patients with metastatic non-small-cell lung cancer (*n* = 100); survey study	To examine current referral practices, timing of referral, beliefs about early palliative care, and patient- and provider-reported barriers.	Oncologists reported referring a mean of 19% of patients at diagnosis, although 83% believed that evidence supported early palliative care. Barriers included absence of symptoms, confidence in managing needs without referral, concern about additional appointments or treatment burden, concern that referral might not be well received, and long waiting times. Many patients had limited knowledge of palliative care but expressed interest in referral.	Despite recognition of the benefits of early palliative care, referral at diagnosis was uncommon. Improved communication, education, and referral pathways may support earlier referral.	United States	III/B
Johnson et al. (2011)	Palliative care	Qualitative study; telephone interviews	General practitioners and cancer specialists (*n* = 40)	To describe physicians' perceptions of facilitators and barriers to referral to palliative care.	Referral decisions were influenced by disease, treatment, and psychosocial characteristics; patient-provider communication and relationships; health-service resources; professionals' skills; and timing of access. Symptom presence, complexity, and intensity, comorbidities, and clinicians' ability to manage symptoms were central considerations.	Referral should consider the combined physical, psychological, psychosocial, and quality-of-life needs of patients, as well as service and communication factors.	Australia	III/A
Schenker et al. (2014)	Palliative care	Multisite qualitative study; semi-structured interviews	Medical oncologists (*n* = 74) affiliated with outpatient oncology practices and palliative care services	To explore oncologist-related factors influencing referral to outpatient palliative care.	Barriers included persistent misconceptions that palliative care conflicts with cancer treatment, the belief that oncologists should manage palliative needs themselves, and limited awareness of locally available services.	Referral may be improved by increasing awareness of the complementary role of palliative care and by strengthening integration and collaboration between oncology and palliative care.	United States	III/A/B
Weaver et al. (2018)	Palliative care	Quantitative cross-sectional online survey	Pediatric oncology settings providing clinical care to children with cancer (*n* = 142), representing 18 countries and 19 US states	To describe pediatric palliative care program structures and services and identify implementation successes and gaps.	The most frequently reported barrier was the perception that pediatric oncologists already provided adequate palliative care. Late referral was the second most frequently reported barrier in centers with pediatric oncology fellowship programs.	Palliative care should be integrated into comprehensive pediatric oncology care, with attention to referral timing and clinician education.	International/United States	III/A
Lee et al. (2021)	Palliative care	Quantitative retrospective cohort study	Hospital admissions of patients with advanced cancer (*n* = 204,175), national electronic databases	To examine racial disparities in the use of inpatient palliative care consultation among hospitalized patients with advanced cancer.	Black patients were significantly less likely than White patients to receive inpatient palliative care consultation.	Race and ethnicity were associated with inpatient palliative care use. Clinicians and systems should address inequities in access to consultation.	United States	III/B
Silva et al. (2020)	Palliative care	Quantitative retrospective cohort study	Patients with colorectal cancer (*n* = 415), hospital cancer unit	To identify factors associated with referral to an exclusive palliative care unit.	Referral was associated with advanced cancer stage (III–IV), malnutrition, and increased systemic inflammation.	Advanced disease and systemic inflammation were associated with referral; these factors characterize palliative care referral patterns rather than general rehabilitation criteria.	Brazil	III/A
Low et al. (2018)	Palliative care	Mixed-methods study	Adults with cancer who were referred to palliative care and died during 2014–2015 (*n* = 595), Uganda Cancer Institute; quantitative records and qualitative clinician data	To describe palliative care referral rates and factors associated with referral among patients with cancer in Uganda.	Patients with poorer Eastern Cooperative Oncology Group (ECOG) performance status were more likely to be referred, consistent with clinicians' description that primarily the sickest patients were referred. Staffing constraints, cultural barriers, and limited interservice communication also affected referral.	Greater integration and collaboration are needed to support timely palliative care referral in settings where patients frequently present with advanced disease.	Uganda	III/A
Wadhwa et al. (2018)	Palliative care	Quantitative retrospective chart review	Patients with advanced cancer referred to an outpatient palliative care clinic (*n* = 337), Princess Margaret Cancer Center	To identify factors associated with early referral and describe symptom severity according to referral timing.	Earlier referral was associated with breast cancer, a disease duration longer than 2 years, chemotherapy for advanced disease, and referral for symptom management. Patients referred later had greater overall symptom burden, including tiredness, nausea, drowsiness, poor appetite, and reduced wellbeing.	Combining symptom screening with timely referral may improve symptom management for people with advanced cancer.	Canada	III/B
Brick et al. (2025)	Allied health rehabilitation	Quantitative secondary analysis of a geriatric-assessment clinical trial; observational cohort analysis	Older adults with advanced cancer and at least one geriatric-assessment-defined physical performance or functional impairment (*n* = 265)	To identify predictors of geriatric-assessment-driven rehabilitation referral and examine associations between referral and function, health-related quality of life, and survival.	Impaired cognition, poorer functional status, and monoclonal antibody treatment were associated with higher odds of referral; polypharmacy was associated with lower odds. Referral was associated with lower odds of decline in activities of daily living and instrumental activities of daily living but was not independently associated with health-related quality of life or survival.	Clinical and treatment factors influenced referral. Further research should examine rehabilitation timing, dose, and actual service use after referral.	United States	III/B
Capozzi et al. (2023)	Allied health rehabilitation	Mixed-methods descriptive feasibility and implementation study	Adults with malignant or benign primary brain tumors referred within the Alberta Cancer Exercise–Neuro-Oncology pathway; 55 patients attended the rehabilitation triage clinic. Qualitative interviews included patients (*n* = 20) and clinical team members (*n* = 5); Calgary, Alberta	To assess rehabilitation and exercise needs and examine the feasibility of a rehabilitation and exercise triage clinic for patients with primary brain tumors.	The study referral rate was 31%, enrollment was 61%, and triage-clinic attendance was 94.7%. Neurological deficits were identified in 51 of 55 attendees; 22 participants required additional rehabilitation referrals. Clinical barriers included forgetting to refer and uncertainty about who was appropriate for referral. Participants and clinicians considered the triage clinic acceptable and useful.	Clinic-based screening and a single triage pathway may improve identification of functional impairment and facilitate referral to exercise, physiotherapy, occupational therapy, or physiatry.	Canada	III/B
Loubani et al. (2024)	Allied health rehabilitation	Qualitative study	Cancer-care professionals, nurses, and social workers (*n* = 28)	To explore barriers and facilitators to occupational therapy referral in cancer care.	Barriers included limited knowledge of the functional impact of cancer-related symptoms, insufficient understanding of occupational therapy's role, organizational and administrative barriers, and limited service availability. Facilitators included collaboration with occupational therapists and awareness of service availability.	Education, stronger interdisciplinary collaboration, and organizational changes are needed to improve occupational therapy referral.	Israel	III/A/B
Maiwald et al. (2022)	Allied health rehabilitation	Quantitative multicenter cross-sectional online survey	Healthcare professionals (*n* = 606), including physicians, social workers, nurses, and psychologists from hospitals, outpatient practices, and cancer counseling centers	To examine professional-, patient-, application-, and organizational-level barriers to the use of cancer rehabilitation.	Barriers included professionals' low valuation of rehabilitation, insufficient coordination, complex application procedures, patient eligibility and expectations, social responsibilities and coping styles, challenges for people with migrant backgrounds in understanding applications, and difficulty admitting patients with multimorbidity.	Access may be improved by addressing professional awareness, coordination, application procedures, and organizational barriers.	Germany	III/A
Marshall et al. (2020)	Allied health rehabilitation	Mixed-methods consensus study using Grid-Enabled Measures and qualitative discussion	Clinicians, researchers, and administrators representing stakeholder organizations (*n* = 166), online consensus workspace	To reach consensus on measures that could identify oncology-related symptoms and functional problems and support referral to appropriate services.	Highly rated comprehensive measures included the National Comprehensive Cancer Network Distress Thermometer, the James Supportive Care Screening, and the Functional Assessment of Cancer Therapy–General. Participants emphasized that screening measures should identify problems and link clinicians to referral algorithms.	Comprehensive screening linked to clear referral algorithms may support identification and referral of patients with rehabilitation and supportive-care needs.	United States	III/A
Moustsen et al. (2015)	Allied health rehabilitation	Quantitative population-based cohort study	People with a primary cancer diagnosis (*n* = 13,840), Copenhagen Municipality	To examine associations between socioeconomic position and referral to cancer rehabilitation.	Referral was more likely among people with higher education, women, single people, people with young children at home, and younger people.	Higher educational attainment was associated with a greater probability of referral, indicating socioeconomic inequity in access.	Denmark	III/A
Nwosu et al. (2012)	Allied health rehabilitation	Quantitative cross-sectional questionnaire survey with free-text responses	Palliative care and respiratory multidisciplinary-team healthcare professionals (*n* = 59), regional cancer network	To examine professionals' perceptions of rehabilitation for people with lung cancer, referral patterns, and barriers to referral.	Barriers included a limited perceived role for rehabilitation within multidisciplinary care, misconceptions about rehabilitation, insufficient communication and understanding among professionals, limited service availability, long waiting times, and patient expectations.	Knowledge of rehabilitation services, interprofessional communication, and service availability should be improved to support referral.	United Kingdom	III/A
Oldenburg et al. (2020)	Allied health rehabilitation	Quantitative one-group pre–post educational study	Medical students (*n* = 37), educational workshop	To examine the effect of an educational workshop on familiarity with occupational therapy and physical therapy roles and confidence in referral.	The workshop significantly improved students' familiarity with occupational therapy and physical therapy and their confidence in making referrals.	Formal education about rehabilitation professions may strengthen future clinicians' referral knowledge and confidence.	United States	II/A
Oksbjerg Dalton et al. (2019)	Allied health rehabilitation	Quantitative population-based cohort study using registry and municipal rehabilitation data	People diagnosed with cancer in Copenhagen during 2010–2015 (*n* = 13,059); 2,523 were referred to rehabilitation within 2.5 years of diagnosis	To investigate associations between socioeconomic indicators and referral to, and attendance at, cancer rehabilitation.	Socioeconomic inequalities were observed in the transition from cancer treatment to post-treatment rehabilitation. Among referred patients, 81% attended a rehabilitation consultation; education and other socioeconomic factors were associated with referral and attendance.	Rehabilitation needs and preferences should be assessed systematically for all patients, rather than relying solely on discretionary referral.	Denmark	III/B
Sparrow et al. (2023)	Allied health rehabilitation	Quantitative retrospective medical-record review	Children aged birth to 3 years referred to an early childhood clinic at a pediatric comprehensive cancer center (*n* = 134)	To describe access and referral patterns to occupational therapy for infants and toddlers with cancer.	Overall, 83.6% received an occupational therapy evaluation, 88.4% were referred to hospital-based occupational therapy, and 42% were recommended for community- or home-based services. Referral patterns differed by diagnosis, with higher referral among children with brain tumors than among those with hematologic malignancies; age also influenced referral.	Standardized developmental monitoring and referral pathways are needed, particularly for community-based services.	United States	III/B

ECOG, Eastern Cooperative Oncology Group; OT, occupational therapy; PC, palliative care; PLWBC, people living with and beyond cancer; PT, physical therapy; SES, socioeconomic status.

^a^Evidence level and methodological quality were evaluated using the Johns Hopkins Evidence-Based Practice Research Evidence Appraisal Tool. Quantitative studies were assigned a quality grade of A, B, or C. Qualitative studies were classified as A/B (high/good quality) or C (low quality). Mixed-methods studies were assigned a quality grade of A, B, or C following appraisal of the quantitative and qualitative components and their integration. Evidence level and methodological quality were evaluated as distinct dimensions and should not be interpreted as a formal risk-of-bias assessment.

Our review identified four main themes: clinicians' perceptions on rehabilitation; the influence of social determinants of health; individuals' cancer characteristics and demographics; and interdisciplinary coordination, communication, and education. Within each theme, findings concerning palliative care were distinguished from those concerning cancer rehabilitation or individual allied health services. Convergence across the two contexts is reported only when supported by studies from both groups. The identified facilitators and barriers were imbalanced, with more barriers reported.

### Theme 1: clinicians' perceptions of referral and services

In the palliative care studies, oncologists' perceptions of the purpose and timing of referral influenced referral practices. Reported barriers included the belief that oncology teams were already providing adequate palliative care, concerns that additional appointments could burden patients, limited familiarity with available services, and concern that referral could be interpreted as abandonment or as a transition to end-of-life care (Feld et al., 2019; Johnson et al., 2011; Schenker et al., 2014; Weaver et al., 2018). In the rehabilitation and allied health studies, referral was similarly affected by limited awareness of rehabilitation services and insufficient appreciation of their role, including OT, PT, and multidisciplinary rehabilitation (Loubani et al., 2024; Nwosu et al., 2012; Maiwald et al., 2022). Although clinician knowledge was relevant in both contexts, the underlying concern differed: palliative care studies emphasized service identity and timing, whereas rehabilitation studies emphasized awareness of functional needs and available services.

### Theme 2: influence of social determinants of health

Social determinants influenced referral and service use in both contexts, but the reported associations differed. In palliative care, one study identified racial disparities in inpatient consultation, with Black patients less likely than White patients to receive a consultation (Lee et al., 2021). In cancer rehabilitation, higher educational attainment was associated with a greater likelihood of referral, and women and younger individuals were more likely to be referred in one population-based study. Socioeconomic disadvantage was also associated with lower referral to, and attendance at, rehabilitation services (Moustsen et al., 2015; Oksbjerg Dalton et al., 2019). These findings indicate inequities across referral pathways but should not be interpreted as evidence that the same determinants operate in the same manner in palliative care and rehabilitation.

### Theme 3: individual cancer characteristics and demographics

In the palliative care studies, referral was associated with clinical indicators of disease burden and complexity. These included advanced cancer stage, exacerbated systemic inflammation, worse performance status, longer time since diagnosis, and referral for symptom management. Symptoms considered relevant to referral included pain, nausea or vomiting, gastrointestinal difficulties, and dyspnea (Johnson et al., 2011; Silva et al., 2020; Low et al., 2018; Wadhwa et al., 2018). These findings describe factors associated with palliative care referral and should not be generalized as criteria for referral to rehabilitation.

In the rehabilitation and allied health studies, different clinical and demographic factors were associated with referral. Among older adults with advanced cancer, impaired cognition, worse functional status, and monoclonal antibody use were associated with higher odds of rehabilitation referral, whereas polypharmacy was associated with lower odds (Brick et al., 2025). In pediatric oncology, children with brain tumors were more likely than children with other cancer diagnoses to receive OT referrals, and referral patterns also varied by age at diagnosis (Sparrow et al., 2023).

### Theme 4: interdisciplinary coordination, communication, and education

Across palliative care studies, referral was affected by communication between oncology and palliative care teams, service availability, staffing limitations, misconceptions about palliative care, and uncertainty regarding shared responsibility for patients' supportive needs (Feld et al., 2019; Johnson et al., 2011; Schenker et al., 2014; Low et al., 2018). Pediatric oncology programs also reported gaps in formal palliative care education and required clinical rotations (Weaver et al., 2018).

In rehabilitation and allied health studies, barriers included insufficient knowledge of the functional benefits of rehabilitation, limited understanding of professional roles, fragmented coordination, long waiting times, and the absence of standardized screening or referral processes (Loubani et al., 2024; Nwosu et al., 2012; Maiwald et al., 2022). Education improved medical students' familiarity with OT and PT and their confidence in making referrals (Oldenburg et al., 2020). Proposed facilitators included comprehensive screening and triage approaches based on physical function, activities of daily living, symptom burden, and quality of life, as well as clearer interdisciplinary referral pathways (Marshall et al., 2020; Capozzi et al., 2023).

Collectively, the findings indicate that referral patterns differed by service context. In the palliative care studies, referral was often late or triggered by advanced disease and symptom burden. In the rehabilitation and allied health studies, referral was inconsistent and depended largely on the recognition of functional needs, professional knowledge, service availability, and established referral pathways.

## Discussion

This scoping review investigated the facilitators and barriers affecting the referral of PLWBC to rehabilitation services across two related but conceptually distinct service contexts: palliative care and allied health rehabilitation services. Palliative care was considered within the broader rehabilitation-related cancer care continuum when studies addressed factors such as symptom burden, functional status, interdisciplinary coordination, service access, or maintenance of quality of life. However, findings concerning palliative care were distinguished from those concerning allied health rehabilitation referral. Across both contexts, referral was often clinician-dependent and affected by limited interdisciplinary coordination, insufficient knowledge of available services, and inequities in access. Nevertheless, the clinical indications, professional perceptions, and referral pathways differed between the two service models. The findings were therefore synthesized around shared themes while retaining this distinction and may inform future clinical and policy efforts to strengthen multidisciplinary cancer care coordination and equitable access to appropriate supportive and rehabilitation services.

The evidence base consisted mainly of Level III studies, including observational, cross-sectional, and qualitative designs. These studies were useful for identifying recurring referral patterns and barriers but provided limited evidence regarding causal relationships. Evidence level and methodological quality were considered distinct dimensions: the Johns Hopkins classification (Crawford, 2009) was used to describe the strength of the study design, whereas methodological quality was evaluated separately. Therefore, the consistency of the identified themes should not be interpreted as evidence of a low risk of bias or a high level of causal certainty.

The included evidence was distributed across two service contexts: eight studies examined referral to palliative care, whereas ten examined cancer rehabilitation, individual allied health services, multidisciplinary rehabilitation, or rehabilitation-related attitudes and education. Across both contexts, the evidence focused primarily on referral barriers rather than on the effectiveness of strategies intended to improve referral or service uptake. However, the clinical factors associated with referral differed between the two contexts. Palliative care referral was more commonly associated with symptom burden, disease stage and complexity, nutritional status, and cognitive or performance status (Silva et al., 2020; Wadhwa et al., 2018), whereas rehabilitation and allied health referral was more closely related to functional needs (Brick et al., 2025; Sparrow et al., 2023; Capozzi et al., 2023), professional awareness, service availability, and the recognition of rehabilitation needs (Loubani et al., 2024; Nwosu et al., 2012; Maiwald et al., 2022). These differences support interpreting the findings within the distinct purposes and referral pathways of each service context (Marshall et al., 2020; Capozzi et al., 2023).

Clinicians' perceptions influenced referral in both service contexts, although the reported concerns differed. In the palliative care studies, oncologists sometimes believed that patients' needs could be addressed by the primary oncology team without palliative care referral. They also expressed concerns that referral might increase the burden on patients or be interpreted as abandonment by the oncology team (Feld et al., 2019; Johnson et al., 2011; Schenker et al., 2014). In the cancer rehabilitation and allied health studies, referral barriers were more commonly related to limited awareness of rehabilitation roles and benefits, uncertainty regarding which patients were appropriate for referral, and insufficient knowledge of available services and referral procedures (Loubani et al., 2024; Nwosu et al., 2012; Maiwald et al., 2022). These findings are consistent with earlier evidence describing the underutilization of rehabilitation services among people with advanced cancer (Cheville et al., 2011). Educational interventions may help address these barriers; for example, education regarding the roles of OT and PT improved medical students' familiarity with these professions and confidence in making appropriate referrals (Oldenburg et al., 2020). Education and training should therefore be tailored to the specific objectives, indications, and referral procedures of palliative care and rehabilitation services rather than addressing them as a single referral pathway.

Social determinants of health influenced referral and service use in both service contexts, although the included studies examined different populations, services, and dimensions of inequality. In palliative care, Black patients with advanced cancer who were discharged alive were less likely than White patients to receive an inpatient palliative care consultation (Lee et al., 2021). In cancer rehabilitation, women, younger patients, and patients with longer education were more likely to be referred to rehabilitation (Moustsen et al., 2015). Socioeconomic differences were also observed after referral: longer education and, in some subgroups, higher income were associated with greater attendance at rehabilitation services (Oksbjerg Dalton et al., 2019). These findings indicate that inequities may arise at different stages of the referral pathway, including referral, acceptance, and subsequent service attendance. However, because the studies were conducted in different healthcare systems and examined distinct services and populations, the magnitude and mechanisms of these inequalities should not be assumed to be equivalent across palliative care and rehabilitation contexts.

The mechanisms underlying these disparities remain unclear and may involve interactions among clinical characteristics, professional decision-making, service availability, financial and organizational barriers, patient preferences, perceived needs, and social roles. Evidence published after the review's search period similarly found that women initiated rehabilitation more frequently than men despite receiving standardized information and administrative support, suggesting that differences in service uptake may persist even when some procedural barriers are reduced (Besserer et al., 2026). This post-search evidence provides contextual support but was not part of the scoping review findings. Previous research has also documented socioeconomic inequalities in cancer rehabilitation and broader disparities across cancer survivorship and advanced cancer care (Haier and Schaefers, 2022; Halpern et al., 2016; Holm et al., 2013; Sampedro Pilegaard et al., 2021). Future research should therefore examine how race and ethnicity, sex and gender, age, socioeconomic position, geography, insurance and reimbursement arrangements, and service availability influence referral and subsequent service uptake. Potential equity-oriented strategies include standardized screening and referral criteria, patient navigation, clinician and patient education, collaboration with community resources, and routine monitoring of referral and attendance patterns across demographic groups (Gupta et al., 2023). However, the effectiveness of these strategies should be evaluated separately within palliative care and rehabilitation pathways and adapted to the organization, funding, and availability of services within each healthcare system.

Clinical characteristics associated with referral differed between the two service contexts. In palliative care, referral or its timing was associated with symptom burden and complexity, comorbidities, poorer Eastern Cooperative Oncology Group performance status, advanced colorectal cancer stage, malnutrition, systemic inflammation, and factors related to cancer type, treatment, and time since diagnosis (Johnson et al., 2011; Silva et al., 2020; Low et al., 2018; Wadhwa et al., 2018). These findings should be interpreted as factors associated with palliative care referral rather than as general criteria for cancer rehabilitation referral. In rehabilitation and allied health services, referral was more closely associated with functional and rehabilitation-related needs. Among older adults with advanced cancer, impaired cognition, poorer functional status, cancer treatment factors, and polypharmacy were associated with geriatric assessment-driven rehabilitation referral patterns (Brick et al., 2025). Among young children with cancer, OT referral patterns differed according to diagnosis and age at diagnosis (Sparrow et al., 2023). Structured screening and triage may facilitate the identification of cancer-related symptoms, supportive care needs, physical impairments, and exercise needs and direct patients to the appropriate service pathway (Marshall et al., 2020; Capozzi et al., 2023). Therefore, routine screening should distinguish between indicators for palliative care referral and functional criteria for rehabilitation or allied health referral. Such an approach may support earlier identification of unmet needs and more consistent referral across the cancer continuum.

Inadequate interdisciplinary coordination and communication emerged as a barrier in both service contexts, although its manifestations differed. In palliative care, barriers included misconceptions about its purpose, uncertainty regarding referral timing, oncologists' perceived responsibility to manage palliative needs themselves, concerns about discussing referrals with patients, limited service availability, and weak coordination with oncology (Feld et al., 2019; Johnson et al., 2011; Schenker et al., 2014; Low et al., 2018). In allied health rehabilitation, barriers included limited communication between oncology and rehabilitation professionals, insufficient understanding of rehabilitation roles, unclear referral responsibilities, fragmented referral procedures, and organizational constraints (Loubani et al., 2024; Nwosu et al., 2012; Maiwald et al., 2022). Conversely, collaboration with rehabilitation professionals and awareness of available services facilitated OT referrals (Loubani et al., 2024). These findings suggest that referral pathways should clarify professional responsibilities and establish communication processes appropriate to the distinct objectives of palliative care and rehabilitation services.

Because the two service contexts have different clinical purposes and referral indications, the following implementation implications relate primarily to cancer rehabilitation and allied health referral pathways. Findings derived from the palliative care studies are used to identify cross-cutting organizational and communication barriers but should not be interpreted as direct rehabilitation referral criteria. From a health systems perspective, the findings suggest that improving rehabilitation referral is not solely a clinical issue but an organizational and policy-level priority. Embedding standardized screening, formalizing referral pathways, and integrating rehabilitation within oncology teams represent potentially actionable strategies; however, their implementation should be adapted to the structure, resources, and referral processes of each healthcare system.

### Limitations

This scoping review has several limitations. First, only English-language publications were included, potentially excluding relevant evidence published in other languages. Despite the use of a comprehensive search strategy, relevant studies may also have been missed because of database coverage, indexing practices, or the terminology used to describe cancer rehabilitation and referral.

Second, most included studies were classified as Level III according to the Johns Hopkins Evidence-Based Practice Model, although their methodological quality was rated as A or B. Thus, the evidence base consisted predominantly of observational, qualitative, and mixed-methods research, with no Level I studies. This limits the ability to draw causal conclusions regarding the factors influencing referral. Furthermore, restricting eligibility to Levels I–III may have resulted in the exclusion of relevant consensus-based and implementation-focused reports, thereby limiting the comprehensiveness of the review.

The heterogeneity of the included studies also limited direct comparison and synthesis. Studies differed in their designs, eligibility criteria, sample sizes, participant populations, settings, referral definitions, and outcome measures. Moreover, the review included evidence addressing referrals to individual allied health services and comprehensive cancer rehabilitation programs, as well as palliative care studies that reported rehabilitation-relevant referral factors. Although these evidence categories were distinguished in the synthesis, differences between their aims, service models, and referral pathways should be considered when interpreting the findings.

Patient perspectives were underrepresented, as relatively few studies directly examined how people living with and beyond cancer perceived referral to and utilization of rehabilitation services. Further research should examine patient-reported facilitators and barriers affecting both referral and service utilization.

Finally, the included studies were conducted across countries with substantially different healthcare systems, cancer rehabilitation delivery models, funding and reimbursement arrangements, service availability, and referral pathways. This cross-national heterogeneity limits the direct comparability of referral patterns and barriers and may restrict the generalizability of the findings. Therefore, the proposed implementation strategies should be adapted to the organizational, financial, and clinical context of each healthcare system.

### Clinical implications for oncology practice

- Functional and supportive-care needs should be routinely screened across the cancer continuum using standardized measures linked to clearly defined referral pathways.
- Referral to cancer rehabilitation should not rely solely on individual clinicians' discretion. Explicit criteria should be incorporated into routine oncology workflows to support consistent identification and referral of PLWBC with functional needs.
- Predefined referral triggers and electronic prompts may support referral decision-making; however, their effects on referral rates, service uptake, and equity require further evaluation.
- Oncology services should routinely monitor referral and attendance patterns across demographic and socioeconomic groups and provide patient navigation or other tailored support when inequities are identified.
- Integrating cancer rehabilitation professionals into oncology teams and clarifying professional roles and communication processes may facilitate earlier referral and improve coordination of survivorship care.
- Referral criteria should distinguish functional indications for cancer rehabilitation from symptom-, disease-, and care-related indications for palliative care.

## Conclusions

This scoping review identified recurring barriers to referral across two related but conceptually distinct service contexts: palliative care and cancer allied health rehabilitation services. In palliative care, referral was often influenced by advanced disease, symptom burden, uncertainty regarding referral timing, and misconceptions about the purpose of palliative care. In cancer allied health rehabilitation services, referral was more dependent on the recognition of functional needs, clinicians' knowledge of rehabilitation roles and available services, and the existence of clear referral pathways. Limited interdisciplinary coordination, clinician-dependent decision-making, and inequities in access were evident across both contexts, although their manifestations and clinical implications differed.

The findings support the development of structured, context-specific referral pathways rather than a single referral model across supportive and rehabilitation services. Within cancer rehabilitation, routine screening of functional needs, explicit referral criteria, and stronger integration of rehabilitation professionals into oncology teams may facilitate earlier and more consistent identification and referral of PLWBC. Within palliative care, efforts should focus on clarifying the purpose and timing of referral and strengthening coordination between oncology and palliative care teams. Because the evidence consisted predominantly of Level III studies and provided limited evaluation of referral interventions, further implementation research is needed to determine whether these strategies improve referral rates, service uptake, functional outcomes, and equity across different healthcare systems.

## Data Availability

The original contributions presented in the study are included in the article. Further inquiries can be directed to the corresponding author.
